# Using spatio-temporal surveillance data to test the infectious environment of children before type 1 diabetes diagnosis

**DOI:** 10.1371/journal.pone.0170658

**Published:** 2017-02-02

**Authors:** Pierre Bougnères, Sophie Le Fur, Isis-Diab collaborative group, Sophie Valtat, Yoichiro Kamatani, Mark Lathrop, Alain-Jacques Valleron

**Affiliations:** 1 Department of Pediatric Endocrinology, Bicêtre Hospital, Paris Sud University, AP-HP, Le Kremlin Bicêtre, France; 2 Inserm U1169, Paris Sud University, Le Kremlin Bicêtre, France; 3 Center for Integrative Medical Sciences, RIKEN, Laboratory for Statistical Analysis, Kanagawa, Japan; 4 Centre National de Génotypage, Evry, France, and Génome Québec Innovation Centre, McGill University, Montréal (Québec), Canada; University of British Columbia, CANADA

## Abstract

The “hygiene hypothesis” postulates that reduced exposure to infections favours the development of autoimmunity and childhood type 1 diabetes (T1D). But on the other side, viruses, notably enteroviruses, are suspected to trigger T1D. The assessment of the possible relationships between infections and T1D still defies the classical tools of epidemiology. We report the methods and results of a geographical approach that maps the addresses of patients to a communicable diseases surveillance database. We mapped the addresses of patients at birth, infancy and T1D diagnosis to the weekly estimates of the regional incidences of 5 frequent communicable diseases routinely collected since 1984 by the French Sentinel network. The pre-diagnostic infectious environment of 3548 patients with T1D diagnosed between 0.5 and 15 years was compared to those of 100 series of age-matched “virtual controls” drawn randomly on the map. Associations were classified as “suggestive” (summer diarrhea, SD, and varicella, V) when p< 0.05, or “significant” (influenza-like infections, ILI) when they passed the Bonferroni correction for FDR. Exposure to ILI and SD were associated with T1D risk, while V seemed protective. In the subset of 2521 patients for which we had genome wide data, we used a case-only approach to search for interactions between SNPs and the infectious environment as defined by the Sentinel database. Two SNPs, rs116624278 and rs77232854, showed significant interaction with exposure to V between 1 and 3 years of life. The infectious associations found should be taken as possible markers of patients’ environment, not as direct causative factors of T1D. They require replication in other populations. The increasing public availability of geographical environmental databases will expand the present approach to map thousands of environmental factors to the lifeline of patients affected by various diseases.

## Introduction

The natural history of T1D makes the search for environmental markers difficult. Indeed, it takes a silent period lasting several months up to several years for the autoimmune reaction to achieve the near-complete destruction of ß-cells, as indicated by the appearance of autoantibodies in a child’s serum long before T1D is diagnosed [1–3]. The rate of ß-cell destruction varies across patients for unknown reasons, but seems accelerated in young children [4]. Environmental factors can affect susceptibility, triggering of autoimmunity, and possibly preclinical course of T1D. The limited concordance of T1D (30–40%) across monozygotic twin pairs is the main proof of major non-genetic risk factors [5–8] that are thought to interact with the genetic background of predisposed children. The genetic background that predisposes to childhood T1D nowadays may not be the same as 30 years ago [9].

The currently increasing incidence of early forms of T1D in many developed countries [10–14] suggests a deleterious remodeling of infants’ environment, either because ancient protective factors have vanished or because exposures causing T1D have emerged or have become more frequent. Incidence and recent increase in incidence of T1D, however, are different across European countries [15].

Infectious exposures are the main environmental suspects, but there is no direct evidence of their implication in T1D risk or natural history in humans. Based on experimental observations in laboratory rodents, the “hygiene hypothesis” postulates that the reduced exposure to infectious agents that children enjoy nowadays has altered the longstanding balance between the developing immune system and microbiological pressure, favoring the occurrence of allergic or autoimmune diseases [16, 17]. Indeed children in developed countries are less exposed to infections than 50 years ago, a result of antibiotics, vaccination, as well as improved hygiene and sanitation. It is thus tempting to think that this may be an underlying cause for the increase in childhood T1D incidence [18, 19]. An inverse relation between increasing T1D incidence and decreasing tuberculosis, rheumatic fever, pinworm infestation, is found at a population level [18, 20, 21], but the almost complete disappearance of these prototypical diseases decades ago cannot explain the more recent increase in childhood T1D. Widespread treatment with antibiotics has decreased the number of infectious episodes while modifying the physiological immune stimulation afforded by intestinal microbiota [22]. Vaccinations, now applied to most infants, prevent measles, mumps, and rubella that affected the vast majority of them in a recent past. On the other side, viral infections may be favored by community-acquired transmission in day-care centers and pre-school nurseries. Rodent models show that viruses may instead induce autoimmune diabetes through their action on ß cells or on the immune system [2, 3, 23–25]. Viruses, notably enteroviruses [26], have been suspected to cause human T1D [27]. Pioneering studies have examined specific viral infections in biological samples from T1D children compared to controls, or have used questionnaires to describe environmental events retrospectively. However, results of these studies are discordant [28–30].

In conclusion, it is possible that early exposure to viruses does protect, or on the contrary trigger T1D, it is also possible that some viral infections protect, while other viruses trigger T1D mechanisms.

The epidemiological search of possible infectious determinants of T1D, and of possible gene-environment associations, is difficult. Indeed, given the low incidence of the disease and its protracted and insidious course [2, 3], cohorts require a long, costly and prospective observation of infectious events in millions of children of the general population. A more promising approach has been developed in the Teddy and BabyDiet cohorts, where specific environmental factors are studied prospectively among children at risk for T1D such as siblings or offspring of T1D patients [31, 32]; those who develop T1D or autoantibodies to ß cell antigens are compared with those remaining disease-free and autoantibody-negative. On the other side, the case-control design requires to retrieve viral events in the past histories of children, with a high risk of recall bias.

An alternative to these classical epidemiological designs can be, as we propose in this paper, to take advantage of the development of the new systems of surveillance of communicable diseases in the general population that provide data with an ever better granularity in time and space (see webpage of the International Society for Disease Surveillance [33] for a list of local and national experiments and resources). Moreover, these databases are increasingly publicly available to researchers without cost. It is then straightforward to map the address of a patient at a given time of his life to know whether he was—or not—living at that time in an area where there was an influenza-like epidemic, or any of the diseases under surveillance. France established a national real time monitoring system called “Sentinel” in 1984 [34], which relies on sentinel general practitioners who report their clinical encounters of 5 frequent communicable diseases. In this work, we show how the geolocation of the patients of a cohort can be used to test if infectious exposures were different in T1D patients and in the general population. This may be done by generating “virtual controls” which provide the reference values. This also enables the search for possible gene-environment associations.

## Patients and methods

### Patients

The patients of this study (n = 3548) were selected from the large multicenter Isis-Diab cohort which was established in 2007 and involves 99 participating centers covering most of continental France. Inclusion criteria for the current study were birth after 01/01/1980 in continental France, Caucasian ethnicity and age at clinical diagnosis between 0.5 and 15 yrs (Table 1). T1D was defined according to the American Diabetes Association [35] and by the presence of at least one class of autoantibodies to glutamic acid decarboxylase, insulin, or islet antigen-2.

**Table 1:** Age distribution of the patients at T1D diagnosis.

	N	Mean (yrs)	Median (yrs)	SD (yrs)	[0–3] yrs (N)	[3–6] yrs (N)	[6–9] yrs (N)	[9–12] yrs (N)	[12–15] yrs (N)
Males	1808	7.2	7.1	3.7	311	425	458	390	223
Females	1740	7.1	7.1	3.6	297	410	437	421	176
Total	3548	7.2	7.1	3.7	608	835	895	811	399

### Ethics

The research protocol was approved by the Ethics committee of Ile de France (DC-2008-693) and the computer security and confidentiality guarantees given to patients was approved by the Commission Nationale Informatique et Libertés (DR-2010-0035). The ClinicalTrial.gov identifier was NCT02212522. All patients provided written informed consent for participation in the study and donation of samples. We obtained written informed consent from the next of kin, caretakers, or guardians on behalf of the children enrolled in the study.

### Geocoding

Addresses of patients at birth, between birth and 4 years and at time of T1D diagnosis were collected as part of an environmental questionnaire filled by patients. Geocoding of the patients’ addresses was done using the ArcGIS 9.3.1 system, the ArcView software, and the database BD ADRESSE® V2 database provided by the French National Geographic Institute (http://professionnels.ign.fr/bdadresse).

### Assessing the environment of patients

Geocoding was used to map the addresses of the patients at the 3 times defined above with public geographical databases, in order to characterize the patient’s infectious exposures from birth up to the clinical onset of T1D.

The weekly infectious exposures of patients to a selected set of frequent communicable diseases were assessed using the French Sentinel System [34] created in 1984, which collects in real time the corresponding information in sentinel general practitioners. Weekly incidence data are available on the web http://www.sentiweb.fr. We studied the past exposure of patients to three communicable diseases of early childhood (measles, mumps and varicella), and to two communicable diseases which affect children of all ages: influenza like illnesses, and acute diarrheas. The weekly surveillance of Influenza-like illnesses started in 1984. The weekly surveillance of measles and mumps–that were very frequent at that time [36]- started at the same date. The weekly surveillance of Varicella and Acute diarrheas started in 1990.

The association of T1D with past infectious exposures was studied using several different definitions of exposure to an infectious environment (see S2 File). We assessed the infectious environment of a child during various time-windows in his life (Table 2) using the regional estimate of the incidences at the places of life. A French region is a Local Administrative Unit of level 1 (LAU1) according to European Union definition (http://ec.europa.eu/eurostat/web/nuts/local-administrative-units); France is divided into 21 regions. We used 2 definitions of the environmental exposures of the children that abstracted the time series of the local incidences during the window: the total of the incidences (noted “cumulative” in Table 2), and the mean of the 10 largest incidences to focus on the epidemic patterns (noted “high” in Table 2). For influenza like illnesses, and acute diarrheas, we studied separately the winter and summer exposures, as influenza occurs only in winter, and as acute diarrheas in winter and summer have different etiologies (Table 2) [37, 38].

**Table 2:** Variables used for the study of the Infectious environment (INF-E). The past exposure of patients was studied for measles, mumps, varicella, influenza like illnesses, and acute diarrheas during different exposure windows. It was abstracted using two methods: “cumulative” is the total of the regional incidences around the case during the window. “high” is the mean of the 10 largest regional incidences to which the patients or the VC were exposed. See Material and Methods, and S2 File for details on the measures of environmental infectious exposures.

Exposure window (method)	Influenza like illnesses exposure	Measles exposure	Mumps exposure	Varicella exposure	Acute diarrheas exposure
**6 months-1yr (“cumulative”)**	INF-E1	INF-E21	INF-E33	INF-E45	INF-E57
**Birth-1yr (“cumulative”)**	INF-E2	INF-E22	INF-E34	INF-E46	INF-E58
**1yr-2yrs (“cumulative”)**	INF-E3	INF-E23	INF-E35	INF-E47	INF-E59
**2yrs-3yrs (“cumulative”)**	INF-E4	INF-E24	INF-E36	INF-E48	INF-E60
**3yrs-4yrs (“cumulative”)**	INF-E5	INF-E25	INF-E37	INF-E49	INF-E61
**1yr-T1D diagnosis (“cumulative”)**	INF-E6	INF-E26	INF-E38	INF-E50	INF-E62
**6 months-1yr (“high”)**	INF-E7	INF-E27	INF-E39	INF-E51	INF-E63
**Birth-1yr (“high”)**	INF-E8	INF-E28	INF-E40	INF-E52	INF-E64
**1yr-2yrs (“high”)**	INF-E9	INF-E29	INF-E41	INF-E53	INF-E65
**2yrs-3yrs (“high”)**	INF-E10	INF-E30	INF-E42	INF-E54	INF-E66
**3yrs-4yrs (“high”)**	INF-E11	INF-E31	INF-E43	INF-E55	INF-E67
**1yr-T1D diagnosis (“high”)**	INF-E12	INF-E32	INF-E44	INF-E56	INF-E68
**Birth-1yr (winter, “cumulative”)**	INF-E13	-	-	-	INF-E69
**Birth-1yr (summer, “cumulative”)**	INF-E14	-	-	-	INF-E70
**1yr-2yrs (winter,“cumulative”)**	INF-E15	-	-	-	INF-E71
**1yr-2yrs (summer, “cumulative”)**	INF-E16	-	-	-	INF-E72
**2yrs-3yrs (winter,“cumulative”)**	INF-E17	-	-	-	INF-E73
**2yrs-3yrs (summer, “cumulative”)**	INF-E18	-	-	-	INF-E74
**3yrs-4yrs (winter,“cumulative”)**	INF-E19	-	-	-	INF-E75
**3yrs-4yrs (summer, “cumulative”)**	INF-E20	-	-	-	INF-E76

### Genome wide genotyping and imputation

A subset of 2521 patients from the Isis-Diab cohort had a whole-genome scan (age at diagnosis: 6.9 ± 3.5 yrs SD). Genotyping was performed at the Centre National de Génotypage (Evry, France) and interpreted at the Genome Québec Innovation Center (Montréal, Canada) with SNP imputation performed at the Riken Institute (Kanagawa, Japan). Technical details are provided in the S1 File.

### Design

#### Environmental study

We used a case-control design with a 1:1 matching in which controls are “virtual controls” (VC), not real persons. The past infectious environment of the T1D patients was compared to that of these VCs. To define the VCs, we followed the key epidemiological principle which is that controls should be obtained from the same source population as the cases [39]. The difficulty to overcome was that the Isis-Diab cohort does not cover uniformly the whole French territory, and that its coverage has varied with time: in the initial years of the cohort, patients were recruited by the first cooperating centers in a much narrower territory than the current one (which is almost all France). To overcome this difficulty, we performed the VC sampling algorithm within successive 2-year classes of birth (starting in 1941, ending in 2011).

VC sampling algorithm: The French territory was partitioned using a grid of 20 x 20 = 400 cells of width of 0.7425° longitude, and height of 0.495° latitude (see S1 Fig). To model the time variation of the recruitment of patients, we considered the spatial distribution of the cohort by classes of 2 years of birth. For each period of two years, we denoted b_*i*_ the number of patients in the cohort who were born in grid cell *i*, and B the sum over all grid cells namely B = Σ(b_*i*_). Thus B is the number of patients of the cohort born in the period of 2 years of birth to which belongs the case for which a virtual control is searched, and b_*i*_ describes the spatial dispersion of the cases of the cohort at that period. The weight of a square *i* in the cohort is w_*i*_ = b_*i*_ /B. A small w_i_ indicates that few patients of the cohort were born in cell *i* during the 2 years period considered. The variation of the w_*i*_ with time models the temporal and spatial variation of the recruitment of patients.

For each case in the cohort, an age-matched VC was sampled thanks to a two-step process. In the first step, the grid cell *i* containing the VC was randomly chosen with probability w_*i*_. In the second step, the precise geographical coordinates of the VC within this “large” grid cell *i* is obtained by sampling a “small” 200m x 200m elementary unit within cell *i*. This sampling was done using the Proportional Per Size R package by Jack G. Gambino (Functions for PPS sampling: http://CRANR-projectorg/package=pps). The local density of population needed by the pps algorithm was obtained at the 200m x 200m precision level from the French National Institute of Statistics (INSEE) 2009 database (https://www.insee.fr/fr/statistiques/2520034). Finally, the age of the VC was taken randomly (uniform distribution) between the age of the case—6 months, and the age of the case + 6 months.

This algorithm was repeated 3548 times to obtain a first set of 3548 VCs. Then the whole process was reapeated 100 times, in order to generate 100 sets of 3548 VCs. The environmental variables were then compared between the group of 3548 patients and each of the 100 groups of 3548 VCs.

### Gene environment analysis

The possibility of gene-infectious environment (GxE) interactions was tested with a case-only method described by Khoury [40] where the exposures to the considered environmental factors are compared among the three genotypic groups at each SNP position.

### Statistics

In the environmental study, exposures of cases and controls were compared with the conditional logistic regression R-package CLogit. A test was reported as *indicative of a possible difference* when the median p value computed over the 100 comparisons of the cases with the 100 sets of virtual controls was <0.05. It was reported as *significant* when the median p value was below the Bonferroni limit p<0.05/N, where N = 76 (total number of exposure variables in Table 2).

In the gene-environment study, we used the case-only method as implemented in PLINK [41]. We first searched for GxE association by using Wald tests based on the linear regression model. Results were visualized using a Manhattan plot (using ggplot2 package in R). Dominant, recessive, and genotypic analyses were performed. A test was considered as indicative of a possible association when p was between 0.05/N and 1/N, and significant when p<0.05/N where N = 7,329,768 (total number of imputed SNPs).

## Results

### Environmental study

**Fig 1:**
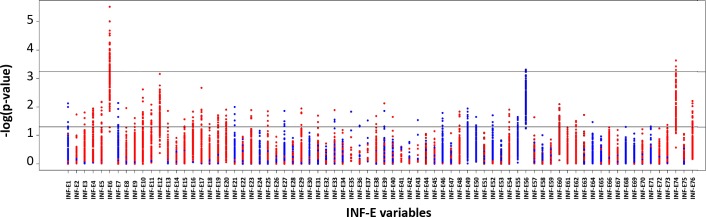
Analysis of the infectious environment of T1D in the Isis-Diab cohort compared with virtual controls. Red/ blue dots indicate that the patients were more/less exposed than the controls. The two lines stand for tests significance at the 0.05 level without (bottom line) and with (upper line) Bonferroni correction for multiple testing (see Table 2 for the definition of INF-E variables).

Among the variables that we used to measure the potential exposure to infectious environment, the total influenza-like illnesses (ILI) burden between the age of 1 year and time of diagnosis was found significantly larger in T1D children than in controls after Bonferroni correction (Fig 1). T1D children also showed a significantly greater past exposure to acute summer diarrheas, and a lower past exposure to varicella, but these differences did not survive the Bonferroni correction (Fig 1).

### Gene-infectious exposures

**Fig 2:**
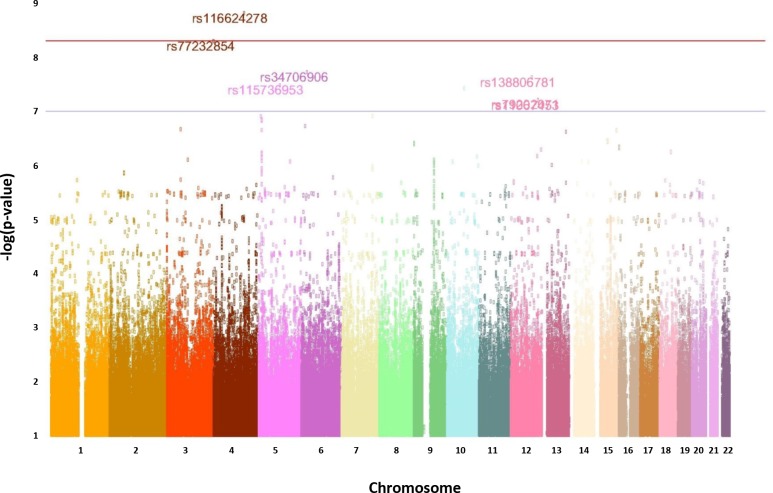
Manhattan Plot showing the SNPs associated with an environmental exposure to varicella. The exposure is the cumulated incidence of chickenpox before age 3yrs computed at the place of birth. The grey line indicates the significance level (p = 1.4/10^−8^) considered as possibly indicating an association at birth. The two SNPs above the red line (located in Chromosome 4) passed the Bonferroni correction (p<7.1 x 10^−9^).

When the infectious exposures were assessed between 1 and 3 years (the child being no longer covered by maternal antibodies), there was an indication of a possible interaction between genetics and infectious exposure. Associations of 2, 1 and 10 SNPs was found with acute diarrheas, ILI, and varicella respectively (Table 3). rs116624278 and rs77232854 survived the Bonferroni correction for an interaction with varicella (Fig 2). rs116624278 is an intergenic SNP located between *PGRMC2* and *JADE1* genes, in a QTL for chronic obstructive pulmonary disease, heart rate and osteoarthritis. rs77232854 is an intergenic SNP located between *PIGG* and *PDE6B*. Both SNPs seem to be in inactive chromatin regions. When cumulative exposures to infectious diseases were assessed over the entire period of 0 to 3 years of age, there was an indication of a possible association for 7 SNPs located on chromosome 11 and for 6 SNPs located on chromosome 16 with acute diarrheas (Table 3). Two other SNPs showed association with varicella, and 5 SNPs with ILI, but none of these associations survived the Bonferroni correction.

**Table 3:** SNPs found associated with an environmental exposure to infectious diseases. The SNPs listed are those for which the p-value of the final test was classified as « indicative » of a possible association (p<10^−7^, see Material and Methods). The 2 SNPs that survived the Bonferroni correction are indicated in bold.

**Infectious environment Disease**	**Exposure window**	**Chr**	**SNP**	**Model**	**P-value (unadjusted)**	**P-value (Bonferroni)**
**Acute Diarrheas**	**1yr– 3yrs**	11	rs7480811	Recessive	4.57E-8	0.3350
		11	rs35611816	Recessive	1.34E-7	0.9839
	**Birth– 3yrs**	16	rs112398774	General	7.99E-8	0.5858
		16	rs73500787	General	1.13E-7	0.8273
		16	rs7191886	General	1.16E-7	0.8491
		16	rs112398774	Dominant	5.07E-8	0.3714
		16	rs73500787	Dominant	7.43E-8	0.5448
		16	rs7191886	Dominant	7.92E-8	0.5806
		11	rs7480811	Recessive	2.35E-8	0.1719
		11	rs35611816	Recessive	4.25E-8	0.3111
		11	rs34123790	Recessive	4.58E-8	0.3354
		11	rs570887	Recessive	9.05E-8	0.6636
		11	rs503156	Recessive	9.86E-8	0.7225
		11	rs535752	Recessive	9.86E-8	0.7225
		11	rs491618	Recessive	1.06E-7	0.7757
**ILI**	**1yr– 3yrs**	15	rs77378263	Recessive	3.06E-8	0.2240
	**Birth– 3yrs**	5	rs115236876	General	1.24E-7	0.9107
		6	rs149303805	Dominant	1.07E-7	0.7871
		15	rs3759862	Recessive	3.27E-8	0.2400
		9	rs11788668	Recessive	4.69E-8	0.3434
		4	rs56089258	Recessive	8.15E-8	0.5971
**Chickenpox**	**1yr– 3yrs**	**4**	**rs116624278**	**Recessive**	**1.55E-9**	**0.0114**
		**4**	**rs77232854**	**Recessive**	**5.13E-9**	**0.0376**
		6	rs34706906	Recessive	1.92E-8	0.1408
		12	rs138806781	Recessive	2.40E-8	0.1759
		5	rs115736953	Recessive	3.35E-8	0.2455
		10	chr10:73778256:I	Recessive	3.87E-8	0.2833
		12	rs79202071	Recessive	6.23E-8	0.4569
		12	rs11067453	Recessive	6.39E-8	0.4680
		7	rs3847106	Recessive	1.24E-7	0.9100
		5	rs140631408	Recessive	1.27E-7	0.9316
	**Birth– 3yrs**	7	rs371617	Recessive	3.87E-8	0.2837
		7	rs445489	Recessive	3.92E-08	0.2873

## Discussion

This study detected differences in infectious exposures between future T1D patients and control children that occurred at the location of residence, while social environment was comparable. This observation supports the existence of a relationship between infections and T1D occurrence. A weakness of our study is that it is exclusively based on addresses of residence and brings no proof that the child living in the area of residence of infected individuals has actually developed the infection reported by the sentinel surveillance network, or even has been exposed to infected individuals. This is why in this manuscript said “exposures” shoud be understood as “potential exposures”.

Our results suggest that T1D is associated with an exposure to more influenza-like illnesses and summer diarrhea during infancy, and to less varicella. These inverse relationships of T1D with different infectious exposures do not support a simple view based on the hygiene hypothesis alone [18]. The development of autoimmunity and T1D may be favored by the interaction of diarrhea with enteroviruses [26] or other members of the gut microbiome [42]. Outside of the gut, T1D may be favored by the previous interaction of viruses with the immune system [23]. For varicella, recent data regarding the presence of the VZV in the neurons innervating the islets of Langerhans could be used to speculate on a protective mechanism that may result from varicella infection [43, 44]. However, we think that one should resist the temptation to extrapolate biological interpretations from purely statistical observations, unless clear mechanisms can support this speculation, which is not the case here.

It would be very interesting, if feasible, to test if the appearance of autoantibodies to ß-cell antigens correlates with infectious exposures, especially because it would make it possible to estimate the time interval between exposure and the earlier detectable manifestation of autoimmunity to ß cells. Indeed, the natural history of childhood T1D studied in siblings and offspring of patients with T1D indicates that autoimmunity often declares itself in early childhood [2, 3]. These observations inspired our exploratory approach by focusing our environmental search on early times of life.

There is an almost unlimited number of possible combinations that can be analyzed by our spatio-temporal approach. First, any complex combination of environmental factors could be considered. Second, an environmental factor can target its victim at different possible ages: this is the reason why this work has tested different windows of exposure, but more could be easily considered with the same approach. Even though the number of possibilities is daunting, the good news is that it is possible. We can for example study the consequences of an exposure during the 2^nd^ trimester of pregnancy just by matching the dates of this second trimester with the values of the environmental databases at the place of residence of the mother. Finally, this is becoming a simple problem of computing power availability. Everything suggests that the computing power that researchers will be able to use will not be a limit to the ambitions of this kind of research. Until environment can be defined more exhaustively at the individual level, our study should be viewed as an exploratory proof-of-concept approach of a multifactorial disease whose environmental causes are yet largely unknown.

Rappaport and Smith have proposed the “exposome” as a new paradigm in which signatures elicited by environmental factors can be detected in serum or in circulating cells [45]. More specifically, next generation sequencing can be used to search for viruses “blindly” in plasma, a promising approach used in the Teddy cohort [46]. Once significant exposures have been identified in blood, it may be possible to determine their sources in the pre-disease environment. Our approach of children’s exposures could be complementary to this strategy, either by providing suspect factors to future exposome analyses, or by testing exposome-driven information across the environmental landscape. The blood exposome and the external exposures can be combined in a cohort or in a case-control approach. More specifically, serology microarrays may soon provide tools for specific pathogen discovery in serum of patients at time of appearance of β-cell antigen antibodies and T1D diagnosis.

The other part of our study was an attempt to cross genetic and environmental information. For this purpose, we used the case-only method proposed by Khoury [40], which compares the level of exposure to the chosen environmental factors across the genotypes at each SNP position. The strong constraint of the case-only approach is that the tested environmental factors need to be independent from genetics. Indeed, if a given child’s infection is influenced by his genotype, a statistical interaction will be found between infection and genotype, which will have no meaning for T1D research. A handful of SNPs were found associated with the studied infectious exposures, indicating possible gene-environment interactions. It is difficult to interpret these statistical observations in the light of a plausible biological interaction between infectious exposures and genetic factors. Notably, the two SNPs that showed a significant interaction with varicella, rs116624278 and rs77232854, were not located within known coding or regulatory sequences of genes directly relevant to immune processes or beta-cell autoimmunity. Their interaction with the varicella exposure eludes our understanding, but might be thought of within a protective context for varicella towards T1D. Our lack of biological understanding of the gene-environment interactions should not be felt as too disappointing since it is common to the vast majority of genomic studies in multifactorial diseases [47, 48]. Our blind genome-wide exploration observed no association of infectious exposures with SNPs located within gene loci known to be involved in infectious or immune response, including HLA class II. This may be due to the lack of involvement of these genes versus the studied exposures, or to the heterogeneity of T1D mechanisms [49]. Indeed, the genetic predisposition to T1D, including HLA genotypes, is known to be highly heterogeneous at the individual level since patients’ genotypes do not carry the same combination of susceptibility alleles. Thus it is possible that particular groups of genes play a role only in specific subsets of T1D patients by interacting with certain environmental factors. If very distinctive of groups of patients, such interaction could escape statistical detection when a whole-genome search is crossed blindly with markers of infectious environment, as performed in the current study.

In conclusion, the current study supports the contribution of infectious exposures to recent childhood T1D epidemiology, yet should be viewed as a methodological proof-of-concept attempt that awaits for confirmation and replication.

## Supporting information

S1 Fig Grid used to generate the virtual controls. The 20x20 grid constitutes approximately a square of 1000 km x 1000 km. Each cell of the grid has a width of 7425° longitude and a height of 0.7425°. France occupies 190 of the 400 cells of the grid.
(DOCX)

S1 File Technical details on genome wide genotyping and imputation. (DOCX)

S2 File Methods used to assess the infectious environment of a patient. (DOCX)
