# Supplementary material for: Using spatio-temporal surveillance data to test the infectious environment of children before type 1 diabetes diagnosis
Source: PLoS One. 2017 Feb 2;12(2):e0170658. doi: 10.1371/journal.pone.0170658 (PMC5289461; doi:10.1371/journal.pone.0170658)

**S1 Fig. Grid used to generate the virtual controls.** The 20x20 grid constitutes approximately a square of 1000 km x 1000 km. Each cell of the grid has a width of 7425° longitude and a height of 0.7425°. France occupies 190 of the 400 cells of the grid.


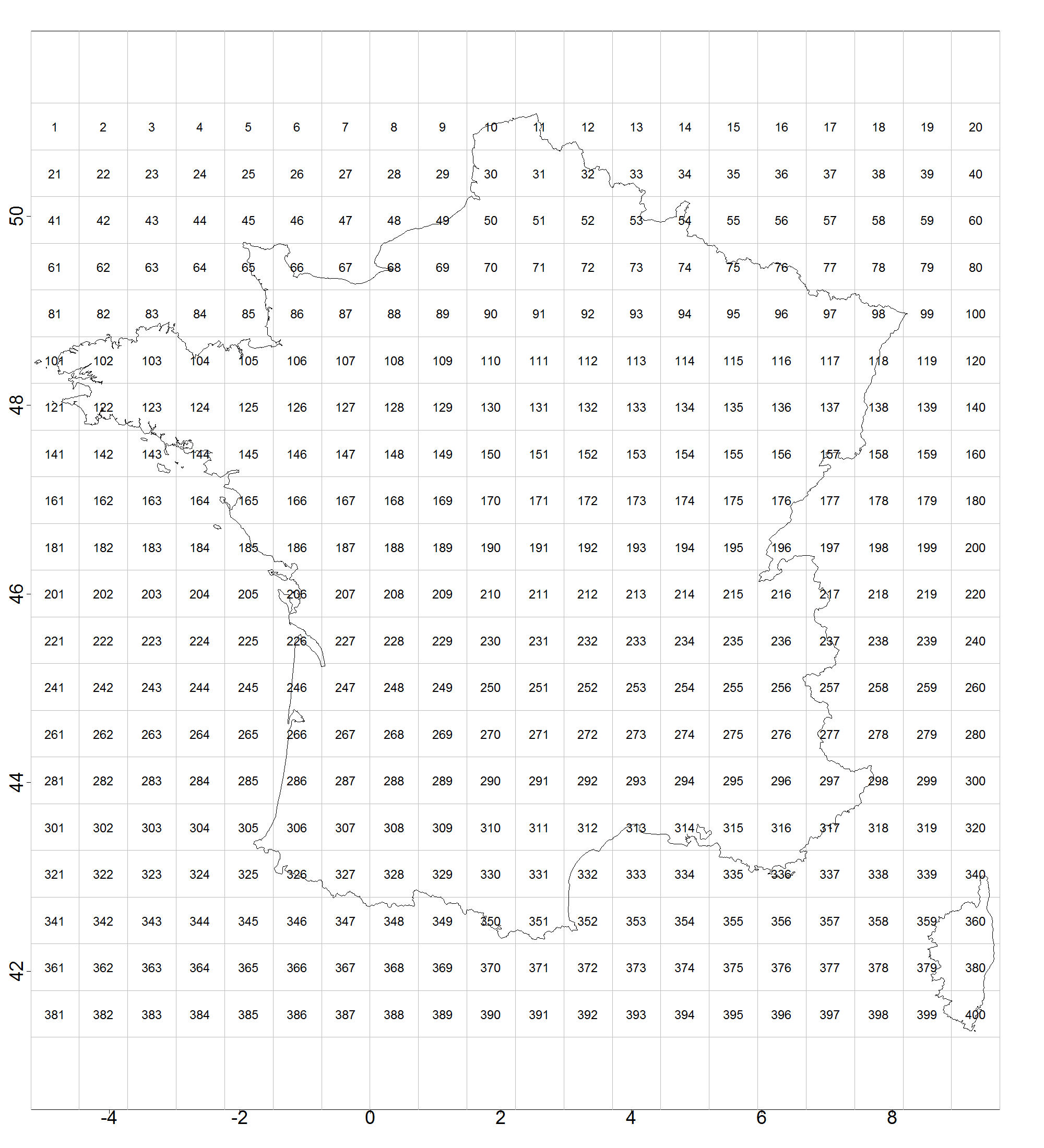

Supplement: S1 Fig — The 20x20 grid constitutes approximately a square of 1000 km x 1000 km. Each cell of the grid has a width of 7425° longitude and a height of 0.7425°. France occupies 190 of the 400 cells of the grid. (DOCX) [file pone.0170658.s001.docx]
