# Supplementary material for: Using spatio-temporal surveillance data to test the infectious environment of children before type 1 diabetes diagnosis
Source: PLoS One. 2017 Feb 2;12(2):e0170658. doi: 10.1371/journal.pone.0170658 (PMC5289461; doi:10.1371/journal.pone.0170658)
Supplement: S1 File — (DOCX) [file pone.0170658.s002.docx]

**S1 File. Technical details on genome wide genotyping and imputation.**

791 patients were genotyped with Illumina HumanCNV370v1_C (370.000 SNPs) microarrays, 838 patients with Illumina Human610-Quadv1_B (610.000 SNPs) microarrays, and 892 patients with Illumina Human Omni 5 Exome microarrays (4.500.000 SNPs). Genome-wide genotyping was performed on bar-coded LIMS (Laboratory Information Management System) tracked samples using three different Illumina microarrays (HumanCNV370v1_C. Human610-Quadv1_B and HumanOMNI5-4v1_B). BeadChips were processed within an automated pipeline as per the manufacturer's instructions. Samples were subject to strict quality control criteria including assessment of concentration, fragmentation and response to PCR. A total of 20 µl of DNA aliquoted to a concentration of 50 ng/µl was used for each array.

In the discovery phase, genome-wide genotypes were used for controlling the quality of the samples. First individuals with call rates <95% or duplicates and individuals who were possibly non-European were removed. By using this filtered sample set, we calculated quality control statistics, and SNPs with call rates <98% or SNPs with a Hardy–Weinberg equilibrium test P–value <1.0 x10^-6^ or SNPs with a minor allele frequency <1% were excluded. Finally, 273,835 experimentally genotyped SNPs (HumanCNV370v1_C), 517,864 SNPs (Human610-Quadv1_B) and 3,309,261 SNPs (HumanOMNI5-4v1_B) were used for imputation analysis. Imputation was done using IMPUTE v2 (<http://mathgen.stats.ox.ac.uk/impute_v2.html>), following the instructions provided by the author ^[[1]](#footnote-1)^. Full sequence data from chromosome 22 from the phase I 1000 Genomes Project was used, and the imputed genetic dosage with R-squared statistics >0.3 was used for the association analysis. As a result, ~30 million SNPs of three Illumina microarray were imputed, and association tests were performed for 7,329,768 SNPs with an imputation quality metric R-squared value of >0.3 and a minor allele frequency of >0.01.

We have used genomic imputation to boost power, fine-map associations and facilitate the combination of results across studies using meta-analysis of our three series of T1D patients that were included in a common standardized genomic database of SNPs that was crossed with environmental data. Genotype imputation has been used widely in the analysis of GWA studies ^[[2]](#footnote-2)^.

1. Howie B, Fuchsberger C, Stephens M, Marchini J, Abecasis GR. Fast and accurate genotype imputation in genome-wide association studies through pre-phasing. Nat Genet. 2012;44(8):955-9. doi: 10.1038/ng.2354. PubMed PMID: 22820512; PubMed Central PMCID: PMCPMC3696580. [↑](#footnote-ref-1)
2. Dahl, A., et al (2016). A multiple-phenotype imputation method for genetic studies. Nature genetics, 48(4), 466-472.

   Marchini, J., & Howie, B. (2010). Genotype imputation for genome-wide association studies. Nature Reviews Genetics, 11(7), 499-511. [↑](#footnote-ref-2)
