# Supplementary material for: Using spatio-temporal surveillance data to test the infectious environment of children before type 1 diabetes diagnosis
Source: PLoS One. 2017 Feb 2;12(2):e0170658. doi: 10.1371/journal.pone.0170658 (PMC5289461; doi:10.1371/journal.pone.0170658)
Supplement: S2 File — (DOCX) [file pone.0170658.s003.docx]

**S2 File. Methods used to assess the infectious environment of a patient.**

We take a first example where all addresses are identical. This is a frequent situation as there is little geographic motility in France.

Marie was born in Paris on July 24^th^ of 1991 and had her T1D diagnosed in Paris on August 13th of 2001.

Using the Sentiweb database, we obtain the weekly incidences of (i) influenza like illness, (ii) measles, (iii) mumps in each French region starting November 1^st^, 1984 and that of (iv) varicella and (v) acute diarrheas starting November 1^st^, 1990.

We take below the example of chickenpox, and we show how the INF-E variables shown in Table 2 were estimated.

Through a sentiweb database query, we obtain (Fig S2.1) the time series of the regional incidence of varicella observed in Marie’s Region between her birth (week 30 of 1991) and her T1D diagnosis (week 33 of 2001).


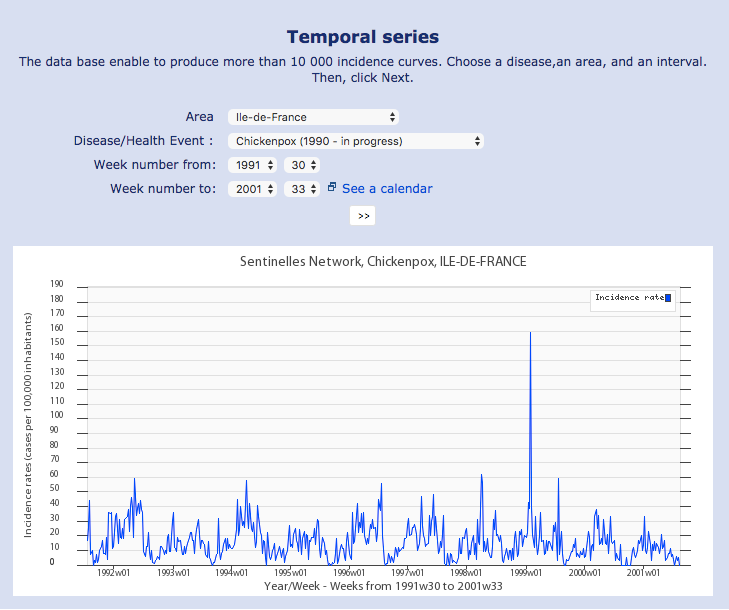


**Fig S2.1. Time-series of the incidence of varicella in the region of Paris where Marie stayed between her birth and her T1D onset.** The sentiweb database can be queried manually at <https://websenti.u707.jussieu.fr/> English version ->Public Health Surveillance -> Database.

We can now compute the different variables shown in Table 2. For example:

- INF-E46 is simply the total of the weekly incidences in Marie’s region between birth (week 30 of 1991) and age 1 year (week 30 of 1992)
- INF-E50 is the total of the weekly incidences in Marie’s region between age 1 (week 30 of 2001) and age 10 (week 33 of 2001) when she was diagnosed with T1D
- INF- E56 is the mean of the 10 largest incidences experienced by Marie between age 1 and age at diagnosis

If the exposure window considered (for instance, from birth to 1 year of age) had taken place before 1988 (for influenza like syndrome) or before 1990 (for chickenpox), the corresponding variables would be set to missing values.

We now consider a more complicated example: Jeanne was born in Paris on July 24^th^ of 1991 (the same day than Marie) but had her T1D diagnosed in Brest (Brittany) on the 1^st^ of August of 2005 (age 14, week 31 of 2005). Her parents declared that her address at age 3 was Paris. Her personal exposure history will be estimated by combining the information on varicella incidence in Paris and in Brest. Between age 0 and 8.5 (midpoint between age 3 and age 14), the personal exposure of Jeanne will be assumed to be related to the incidence in the Paris region, and after age 8.5 (24/01/2000, week 4 of 2001) it will be assumed to be related to the incidence in the Brest region (Fig S2.2). Note that the idea behind this work is that in the near future, the current painful, scarce and imprecise acquisition of data on patients places of life, will become easy with the generalized automatic geolocalization using smartphones and other devices of the Internet Of Things.


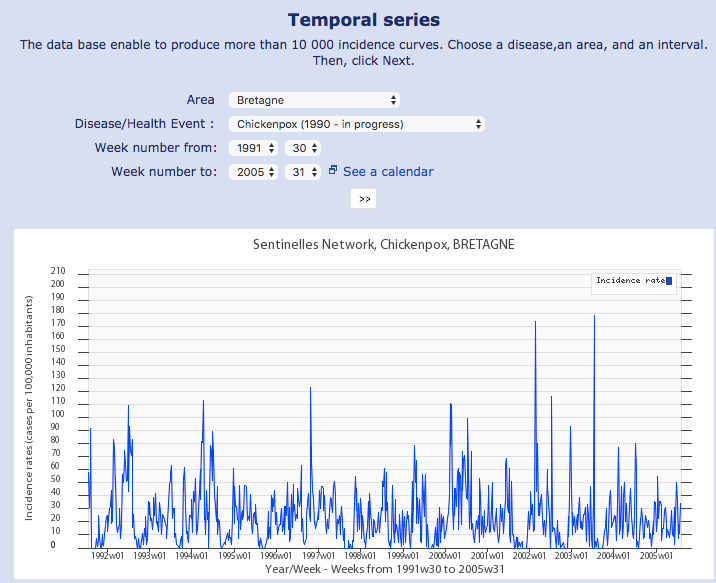

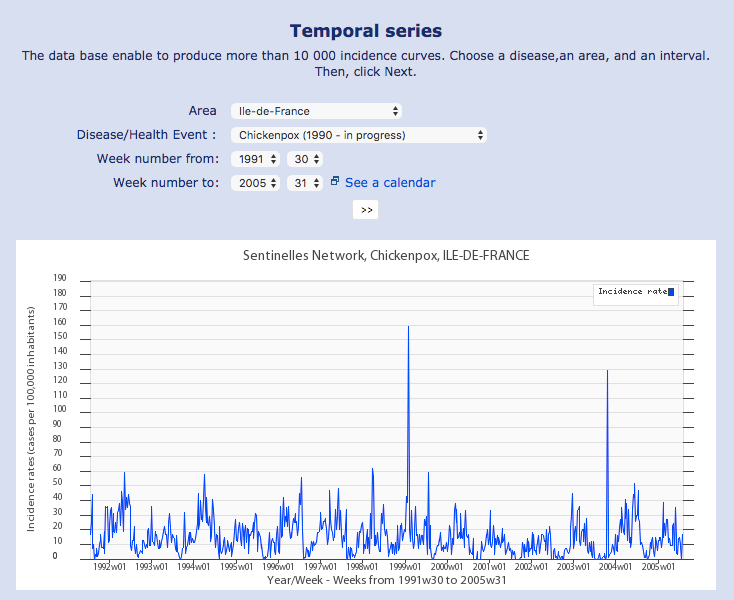


**Fig S2.2. Time-series of the incidence of varicella in the regions of Paris and Brest where Jeanne stayed between her birth and her T1D onset.**
